# RETRACTION: Histopathological Study of Cyclosporine Pulmonary Toxicity in Rats

**DOI:** 10.1155/jt/9757928

**Published:** 2025-09-09

**Authors:** Journal of Toxicology

RETRACTION: S. S. Elshama, A.E-M. EL-Kenawy, and H-E.H. Osman, “Histopathological Study of Cyclosporine Pulmonary Toxicity in Rats,” *Journal of Toxicology* 2016 (2016): 2973274, https://doi.org/10.1155/2016/2973274.

The above article, published online on 28 January 2016 in Wiley Online Library (https://wileyonlinelibrary.com), has been retracted following the agreement of the journal's Chief Editor, You-Cheng Hseu; and John Wiley & Sons Ltd.

The retraction has been agreed following an investigation into concerns originally raised by *Actinopolyspora biskrensis* and *Hoya Camphorifolia* on PubPeer [1], which identified multiple inconsistencies in Figures 4c, 5a, and 5c.

As a result of this investigation, the conclusions of the article are considered unreliable.

The authors have been informed of this retraction but did not provide a response.
